# Environmental Variables in the Mexican Tropics and Their Relationship to Management and Welfare in Crossbreed Zebu Cattle

**DOI:** 10.3390/ani16020288

**Published:** 2026-01-16

**Authors:** Miguel A. Damián Valdez, Virginio Aguirre, Saul Rojas Hernández, Jaime Olivares Pérez, Mariana Pedernera, Abel Villa-Mancera, Lucero Sarabia Salgado, Agustín Olmedo-Juárez, Fredy Quiroz Cardoso, Moises Cipriano Salazar

**Affiliations:** 1 Facultad de Medicina Veterinaria y Zootecnia N°1, Universidad Autónoma de Guerrero, Carretera Nacional Cd. Altamirano-Iguala Km.3 Colonia Querenditas, Cd, Altamirano 40610, Guerrero, Mexico; miguelon3020@hotmail.com (M.A.D.V.); saulrh@hotmail.com (S.R.H.);; 2 Facultad de Ciencias Agropecuarias, Universidad Autónoma del Estado de Morelos, Avenida Universidad 1001 Colonia Chamilpa, Morelos 62210, Cuernavaca, Mexico; avirginio@uaem.mx (V.A.); pedernera@uaem.mx (M.P.); 3 Facultad de Medicina Veterinaria y Zootecnia, Benemérita Universidad Autónoma de Puebla, Tecamachalco 75482, Puebla, Mexico; abel.villa@gmail.com; 4 Instituto Nacional de Investigaciones Forestal, Agrícolas y Pecuarias–CENID-SAI, Cuernavaca 62550, Morelos, Mexico; olmedo.agustin@inifap.gob.mx

**Keywords:** temperature and humidity index, animal welfare, cattle fly, bovine behavior

## Abstract

Most animal welfare (AW) assessment protocols have been developed for intensive production systems and European cattle, raising concerns about their applicability in the tropics. To compare the results obtained by using the welfare quality (WQ) assessment for fattening cattle in the dry tropics, relevant modifications were implemented in 20 cattle production units (PUs) during the dry (DS) and rainy (RS) seasons. Regarding the principle of good feeding, only during the RS, between 20% and 25% of the farms maintained their animals in the acceptable and good categories, compared to the DS, where all PUs were classified as unacceptable (*p* < 0.04). Under the “Appropriate Behavior” principle, only 15% and 60% of the PUs maintained their animals at good and acceptable levels, respectively, in the RS, but not in the DS (*p* < 0.001). Conversely, during the DS, better scores were obtained for the measures and criteria in the Good housing group, with 45%, 50%, and 5% of PU classified as acceptable, good, and excellent, respectively, while for the RS, only 15%, 30%, and 5% reached these levels (*p* < 0.01). Meanwhile, under the “Good Health” principle, better animal health scores were observed during the RS, with 20%, 30%, and 50% of farms classified as acceptable, good, and excellent, compared to the DS, where only 70% and 10% of farms maintained their animals at good and excellent levels (*p* < 0.01). It is concluded that better animal welfare (AW) indicators were recorded during the RS, and the adjustments we applied to the conventional WQ protocol comprised a modification for the criterion that included the prolonged absence of thirst as well as adding six new indicators (measures) to the principles of housing, health, and behavior, which are considered essential for evaluating AW in cattle that are managed under extensive conditions by season.

## Simple Summary

Producing animal-based foods is a challenge for the agricultural sector due to growing human demand. This imbalance between supply and demand has led to an intensification of animal husbandry that is driven solely by economic interests, neglecting animal welfare and the humane treatment of animals raised for food. This document presents a study to measure the welfare conditions in which beef cattle live and are raised on small production units. The methodology used was the welfare quality protocol, adapted to livestock farming on grazing and predominant environmental and management conditions in the two-year seasons (rainy and dry).

## 1. Introduction

Animals require welfare conditions in any production system [1]. However, it is often difficult to assess animal welfare (AW) under different conditions in which they are reared, considering the importance of positive experiences in animals’ lives that contribute to their mental well-being [2,3]. In particular, for cattle, there are no specific protocols for application in extensive, semi-extensive, and semi-intensive production systems. Due to the characteristics of the cattle, the limited handling, and their interaction with humans in these systems, it is difficult to assess AW using a single, general protocol [4]. Given this situation, it is necessary to have an evaluation protocol that provides reliable results on the animal welfare status of livestock in different production systems [5]. Due to the importance of animal welfare, protocols were developed to facilitate its evaluation and allow livestock farmers to make decisions that directly or indirectly improve the living conditions of their animals and their production [3,6]. The most widely used international protocols for assessing animal welfare are the Animal Welfare Indicators (AWIN) developed by the European Union, the protocol proposed by the World Organization for Animal Health (WOAH), and the welfare quality (WQ) assessment protocol [7]. All of these assessment protocols address the animal welfare level by using indicators that are presented as quick, simple, inexpensive, repeatable, objective, and representative measurements. The WQ protocol comprises four principles and 12 criteria, and its applicability has been demonstrated in different countries [1,6,8,9,10]. However, Alejos et al. [11] and Zurita et al. [12] reported some problems when it was used in different livestock systems, which resulted in unreliable evaluations in some criteria. Therefore, the objectives of this work were to evaluate animal welfare by using the WQ protocol in the dry and rainy seasons, in crossbred Zebu cattle raised in a semi-extensive system in a tropical environment, and also to develop adaptations to the protocol for a more objective evaluation of animal welfare under the aforementioned specific conditions.

## 2. Materials and Methods

This study was developed in the tropics of Guerrero, Mexico (18°25′ N and 100°31′ and 100°43′ W), at 250 m above sea level, during two seasons in the 2023 year: the dry season (DS), which covers the months of November to May, and the rainy season (RS), which covers the months of June to October.

### 2.1. Production Units (PUs)

Twenty animal production units (PUs), characterized by a semi-extensive system and dedicated to the production of calves for fattening, were used. The PUs were randomly selected considering the availability of the owners (Free consent letter signed). The number of animals in each PU varied from 15 to 50 cattle, including young, adult, male, and female animals. During the DS phase, the animals were fed on corn (*Zea mays*) or sorghum (*Sorghum vulgare*) stover through grazing, requiring supplementation with commercial feed, ground corn cob, corn, or sorghum silages. In the RS, the animals were managed under grazing conditions on native pastures that included forages such as *Cynodon dactylon*, *Cypresus rotundus*, and *Muhlenbergia macroura*. The animals used were crossbreeds of *Bos indicus* x *B. taurus* cattle (Brahman, Sardo Negro, Gyr) x (Simmental, Charolais, Beefmaster, Brown Swiss, Angus). The same units were used in the RS and DS. Measurements were taken directly in the grazing areas at 10:00 AM (one unit was evaluated per day). All animals in the units were evaluated by five trained researchers during both seasons over a period of 20 consecutive days through rounds to assess the animals in their natural environment.

### 2.2. Evaluation of the WQ Protocol

The WQ protocol considers four principles as follows: good feeding, good housing, good health, and appropriate behavior. Within each principle, it includes evaluation criteria, and within each criterion, measures or indicators of welfare quality (WQ). Based on the welfare quality final scores, the herd welfare level may be classified into one of the four following categories: unacceptable, acceptable, good, and excellent [7].

### 2.3. Adjustments Developed to the WQ Protocol

The use of the welfare quality protocol to evaluate animal welfare in production units required adjustments to some of the indicators. Table 1 shows that the following indicators were not evaluated (omitted): animal cleanliness, escape zone, access to grasslands (days per month, hours per day), and a Qualitative Behavioral Assessment (active, relaxed, nervous, aggressive, indifferent, happy, frustrated). On the contrary, the indicators of respiratory rate (gasping scale), shaded space per animal, presence of flies, branding, placement of earrings, weaning method, and clamping system were added. Only one indicator was proposed for modification (provision of sufficient water and clean water sources).

### 2.4. Good Feeding

#### 2.4.1. Absence of Prolonged Hunger

Body condition was assessed by observing all animals from the back and side, focusing on the back, tail base, vertebrae, and ribs. Animals with prominent bone in the observed regions were considered thin and were classified into one of two categories: 0 for satisfactory or normal condition, or 2 for very thin animals, as outlined in the protocol. The score was calculated by determining the percentage of very thin animals based on the total number of animals in each unit.

#### 2.4.2. Absence of Prolonged Thirst

In the DS, all existing water sources accessible to the animals in the grazing area were counted. Water sources were classified into one of three categories that were indicated by the protocol: 0 for drinking troughs with clean water and drinkers (or drinking troughs), 1 for partially drinkers (or watering devices) but with clean water, and 2 for troughs with dirty water. In the RS, the absence of water troughs was not considered a factor, as the cattle drank from natural water sources (stream, banks, dams, and canals). For this reason, the absence of prolonged thirst was rated by measuring the length of the water sources that the animals could access without difficulty, and then measuring the space available per animal and categorizing it as clean water when there were no unpleasant odors [13].

### 2.5. Good Housing

#### 2.5.1. Animal Cleanliness

Animal cleanliness was assessed by the direct observation of one side of the body and the rear, including the legs and udder. The rating was given as follows: 0 = no soiling, less than 25% of the body dirt plaques, or less than 50% liquid splashing on the animal’s body; 2 = 25% of the animal’s body or more that is covered by dirt plaques or more than 50% liquid dirt splashing. The final rating was calculated by determining the percentage of animals grouped into the categories 0 and 2 described above.

#### 2.5.2. Movement Ease

This was determined by establishing the available space (m^2^) for movement and rest for each animal, considering a range of 2 to 9 m^2^ per animal according to the WQ protocol. The WQ protocol (welfare quality protocol) for animal welfare in grazing systems does not consider the Shaded space per animal indicator as a rest area, as it only considers animals housed in barns. The recommendation for this indicator was to integrate into the protocol a procedure for the evaluation of available natural space with shade for animal shelter, and it was established as a minimum of 2 m^2^ to comply with the standardization by the WQ protocol.

#### 2.5.3. Thermal Comfort

The respiratory rate (gasping scale) indicator—although the WQ protocol does not have a specific measure for respiratory rate—considers the climatic conditions (high temperatures, relative humidity, and solar radiation) to which animals are exposed, which are irregular in grazing areas in the tropics. As such, it is important to have a measure that estimates the conditions of heat stress in animals. To evaluate this indicator, it was proposed to include a panting scale that was adapted from Islam et al. [14], where 0 = normal breathing (50 to 60 exhalations/min); 1 = increased breathing (from 60 to 90 exhalations/min); 2 = moderate panting with little presence of mucus and 90 to 120 exhalations/min; 3 = severe panting, characterized by an open mouth, greater presence of mucus, and 120 to 150 exhalations/min; 4 = severe panting, with an open mouth and protruding tongue, much saliva, and an extended neck. This may group animals into either levels 2, 3, or 4 on the panting scale.

### 2.6. Good Health

#### 2.6.1. Absence of Injuries

The animals were assessed by directly observing them in motion, quantifying the number of animals with some degree of lameness, and classifying them into one of two categories: 0 = animals without lameness and 2 = animals with lameness, and then determining the percentage of animals with hoof lesions. The animals were also examined so as to identify hairlessness areas, lesions, or inflammation on the skin, classifying them into one of three categories: 0 = animals without lesions, 1 = animals with minor lesions, at least one patch of hairlessness, and no inflammation, and 2 = animals with severe lesions.

#### 2.6.2. Disease Absence

Animals exhibiting coughing were identified through direct observation. Classification was performed at the group level, quantifying the average number of coughing episodes per animal over a 15 min period. Nasal discharge was assessed by observing animals for clearly visible light yellow/greenish nasal discharge, assigning 0 = animals without nasal discharge and 2 = animals with evident nasal discharge. Ocular secretion was evaluated by observing wet or dry secretions from the eye of at least 3 cm in length. The percentage of animals with nasal discharge or respiratory problems was also quantified by observing animals with deep, labored breathing, where exhalation was assisted by the abdominal muscles and often accompanied by a pronounced sound. At the group level, the percentage of animals with respiratory difficulties was calculated. The number of animals with diarrhea was determined by counting animals with watery excrement and fecal staining in the perianal area; the percentage of animals with diarrhea was estimated from these records. Tympany was determined by observing the left paralumbar fossa.

The absence of pain induced by handling procedures was assessed using a questionnaire administered to the owners of each production unit. This questionnaire was designed to gather information on handling practices such as dehorning, branding, weaning, and the use of anesthetics or analgesics. Based on the responses, a percentage score was calculated for each group using a decision tree established in the WQ protocol.

#### 2.6.3. Absence of Ectoparasites

The WQ protocol does not consider the absence of ectoparasites criterion for animal welfare. This criterion was evaluated using the presence of flies principle. The counting of the number of flies that land on an animal, using the methodology used by Rojas-Hernández et al. [15], was an indicator of animal welfare. The number of flies that postured on the animal was calculated by counting the scapula-dorsum, legs, belly, and neck on one side and multiplying by two to obtain the total number. The criteria were applied as follows: 0 to 200 flies: low acceptable level for AW; 201 to 500: high level, and 501 to 1000: very extreme, considered to have affected AW.

### 2.7. Appropriate Behavior

#### Social Behaviors Expressed

Agonistic behavior was assessed by observing the animals for ten minutes. Aggressive interactions such as headbutting, displacement, chasing, and fighting were recorded, and the average number of aggressions per animal was calculated for the observation period. Cohesive behavior was also observed, and for ten minutes, the number of grooming and play events was recorded, and the average number of cohesive behaviors per animal was estimated.

### 2.8. Rating of the PUs

The procedure described by our adapted WQ protocol was carried out; the result was expressed as a percentage, and subsequently, the PUs were grouped into four categories based on the overall score for herd welfare level, which were described as excellent when >80 to 100%, as acceptable when >60 to 80%, as good when >20 to 60%, and as not classified when 0 to 20%.

### 2.9. Climate Data: Ambient Temperature, Relative Humidity, Precipitation Level, and THI

During the evaluated periods, ambient temperature and relative humidity were also measured using a Taylor digital brand ambient thermohydrometer. Measurements were recorded daily in the DS and RS. The rainfall data were obtained from the records of the National Water Commission [16]. The THI was estimated with the formula THI = (1.8 × T° + 32) − (0.55 − 0.55 × RH/100) × (1.8 × T° − 26), where THI = the temperature and humidity index, T° = ambient temperature, and RH = relative humidity.

### 2.10. Data Analysis

The data were analyzed using the non-parametric Mann–Whitney U test, comparing animal welfare measurements to the changes in climate variables (temperature, humidity, and precipitation) between the dry and rainy seasons.

## 3. Results

### Animal Welfare Principles Results on Seasons

**Figure 1:**
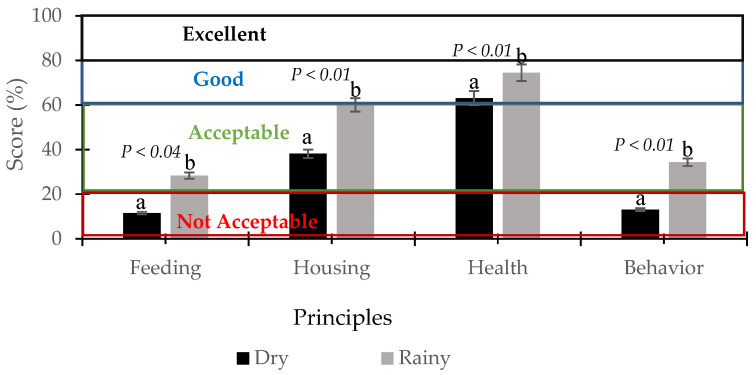
Comparative evaluation of AW by season and classification criteria based on the WQ protocol (average final scores for all 20 PUs; ^a,b^ Express differences in animal welfare in each criteria by year season) in a tropical environment.

In general, the principles of well-being were modified by the season of the year (Figure 1). Good feeding and health principles, as well as the freedom to express appropriate behavior, were better in the RS, since according to the WQ scale in the good feeding principle, the average score of the 20 PUs was 28.3% in the RS compared to 11.7 in the DS (*p* < 0.04), and in health (RS:74.5% vs. DS:63.1%) and in behavior (RS: 34.3% vs. DS: 13.05%) (*p* < 0.01) (Figure 1). For the good housing principle, the highest score was obtained in the DS, with an average of 60%, which was probably related to the changes in animal management between seasons. For example, in the RS, the animals remained on the pastures and were not taken to the stables. These changes are due to the availability of food and water in natural watering holes (limited conditions in the DS), and are attributed to the predominant climatic conditions in the RS, such as lower temperatures (23.4 °C) and higher humidity (90.2%), resulting in more rainfall (561.2 mm) (Table 2), which favored the growth of forage grasses. In general, when all the WQ criteria are considered, it can be concluded that in the DS, the production units were classified as unacceptable because it establishes that when the classification is less than 20% in two of the four criteria, as observed in the DS in Figure 1, the AW is rated as unacceptable in all criteria.

Dry season (DS): The good feeding principle was classified as unacceptable in all production units evaluated. The principle of appropriate behavior was rated as unacceptable in 95% of the production units. Regarding the housing principle, 40% of the production units kept their animals in acceptable facilities, while 60% provided good facilities. The health indicator was considered good for 75% of the cattle production units, excellent in 5%, and unacceptable in 20% (Table 3).

Rainy Season (RS): In the case of the good feed principle, 25% of the production units (PUs) were classified as good feeding, 10% were considered acceptable, and 65% unacceptable. For the species-specific behavior principle, only 15% of the PUs exhibited excellent behavior, 60% were considered acceptable, and only 25% inadequate. Regarding the comfortable housing principle, cattle in 5% of the PUs were housed in excellent conditions, 20% in good conditions, another 20% in acceptable conditions, and 55% of the PUs kept cattle in unacceptable conditions for this indicator. The good health principle was excellent in 50% of the production units because the animals had good body condition, were without signs of mistreatment and were free of disease; in 30% of the production units, the animals were classified as being in good health, and only in 20% the cattle were classified as being in acceptable condition for this indicator (Table 3).

## 4. Discussion

During the DS, animal welfare, according to the principle of good feeding, was classified as unacceptable in all PUs, suggesting that the animals suffered prolonged hunger during this season, which was characterized by extreme living conditions (temperature and zero precipitation) that limit the quantity and quality of food. In this regard, Li et al. [17] and Hernandez et al. [18] indicated that zero forage production during the DS represents a serious problem for livestock feeding. Due to the long drought period, it is necessary to supplement the cattle diet with nutrients that are often not enough to prevent the animals from depleting their nutrient reserves (glycogen, lipids, and muscle proteins) and losing body condition, which can sometimes lead to death [19]. Feeding conditions improved during the RS, where animals had access to a greater quantity and quality of forage with the ability to select a varied diet through direct grazing. This favored the intake and availability of nutrients for maintenance and weight gain, which increased the animals’ body condition. In addition, constant access to natural water sources also contributed to a greater degree of welfare, coinciding with what was reported by Hernandez et al. [18] and Broom [20].

In most of the evaluated farms, there were no roofed or covered facilities for the protection of the animals. In the DS, the cattle were kept clean of feces and had sufficient space to move freely; they were classified as being in acceptable to good health. On the contrary, in the RS, the animals had sufficient space for movement, but the cleanliness of the cattle was classified in the evaluated PUs as unacceptable; however, due to the prevailing climatic conditions in the rainy season, the dirtiness of the animals’ skin should be omitted from the WQ protocol as an applicable valid indicator for Mexico local conditions in this regard.

Health was rated as good and higher in the RS, probably attributed to indicators such as the presence of lesions and inflammation on the animals’ skin that were observed more frequently during the DS because the animals were subjected to smaller spaces when the supplement was provided, and the cattle competed by hitting and injuring others of lower rank or smaller size. This behavior was also reported by Damián et al. [21], who observed that in animals handled in confined and reduced spaces, more agonistic behaviors were generated, which affected their well-being. Conversely, in the RS, the animals were grazed in large areas with abundant shade, which reduced hierarchical aggression and resulted in fewer bodily injuries, similar to the reported by Cooke et al. [22]. Conversely, lameness in the animals tended to be more prevalent in the PUs in the RS, attributed to the higher soil moisture, which causes softening and sensitivity of the hoof with some degree of lameness, which coincided with Rahim & Sobur [23]. The same trend was observed in indicators of the presence of disease symptoms, such as eye and nasal discharge, respiratory distress, or diarrhea, which tended to be higher in the PUs of concern during the RS. It is very likely that the animals’ genotype (*B. indicus*) and their adaptation to environmental conditions (the THI of the DS, 75.2–91.3, and RS, 69.3–90.3) (Table 2) were key factors in the decrease in the presence of diseases when compared to other studies in *Bos taurus* cattle [24]. Regarding the painful handling of animals, it was verified that branding and dehorning activities are carried out mainly and indiscriminately throughout all year-round seasons.

The ambient temperature, combined with the lack of rainfall and low relative humidity during the DS, were factors that contributed to the environmental deterioration conditions during this period, affecting animal welfare compared to those observed in the RS. Table 2 shows that the THI was similar in the DS and RS; however, in the DS, it was the temperature factor that increased the THI, and in the RS, it was the relative humidity factor. Guerra et al. [25] indicated that in very hot environments, heat stress occurs, altering the physiological, productive, and reproductive responses of livestock. Therefore, Avendaño et al. [26] reported that *Bos indicus* cattle have a better capacity for physiological thermoregulation when shaded areas are implemented; however, the prevailing conditions in the DS have a strong impact on feed availability and consumption. The scope obtained in this study had limitations, especially regarding the indicators measured and the variation that the animals could express during a day (24 h) in each indicator due to the THI effects. Animal welfare is very complex, and there may be more indicators related to the topic that were omitted in this study; however, the procedures used can be the basis for more evaluations in tropical areas with similar livestock management and animal genotype.

### Changes Implemented to the Conventional WQ Protocol

Good feeding: The methodology proposed by the WQ protocol in the RS was not adapted to the prolonged absence of thirst criteria because the animals were managed entirely on pasture in areas with natural water sources, such as streams, dams, or ponds, where they have water access. However, with the WQ protocol adaptations, its use became feasible to estimate thirst absence in cattle under grazing conditions in the RS in tropical areas. Lapo et al. [27] also considered it feasible to implement scientifically sound strategies adapted to the local context to provide AW conditions and maintain sustainable productivity. Vargas et al. [28] reported that cattle grazing under tropical conditions face many limitations, mainly feeding and water access, challenging the ability of humans to provide better conditions.

Good housing: The WQ protocol considers the cleanliness of animals as important for their welfare; it believes that animals kept in an intensive production system, where housing leads to the accumulation of feces and urine in resting areas, could be forced to rest on dirty floors. In Table 1, it can be seen that the animal cleanliness indicator was omitted, and, as such, cannot be considered a determinant of AW in the RS conditions of this study. This is due to cattle being raised on pasture in wide areas, with them being exposed to climatic factors (rain) where accumulated dirt on their body may be due to the animals soiling their body with wet soil in wallowing areas as a natural behavior to ward off insects (flies and ticks) to temporarily relieve the discomfort they cause [29], or, alternatively, to cool down and protect themselves from the sun’s rays [30]. With the addition of the respiratory rate and shade area indicators, with the measurement scales proposed, it was possible to estimate animal welfare conditions based on the principle of good housing locally for grazing cattle. Romera et al. [3] mentioned that the indicators included in the protocols should be expanded or modified so as to be applicable to local breeding and environmental practices that involve more measures in relation to the five domains. Also, in tropical conditions, high temperatures and solar radiation force animals to seek shade to protect themselves from the sun, especially during peak radiation hours— a behavior that was observed in cattle by Salas et al. [4].

Good health: The presence of flies’ indicator, added in the assessment protocol, as seen in Table 1, contributed to evaluating the good health principle because fly populations vary with the seasons due to the prevailing environmental conditions (rain, humidity, and temperature). This indicator can affect weight gain, milk production, and promote disease transmission, anemia, skin damage, and consequently, animal welfare in extensive systems in tropical regions [18,31]. The WQ protocol assesses the use of anesthetics and analgesics during dehorning, castration, and tail docking in intensive systems. However, this study observed that the most common practices were dehorning and other painful or stressful procedures, such as branding, weaning, ear tagging, and restraint methods. These observations constitute limitations in this study, which recommends adjustments to the WQ protocol to include these procedures in other studies.

In terms of appropriate behavior, the WQ protocol estimates that if animals allow an approach of less than 1 m, they are classified as adapted to humans and do not experience stress when people invade their flight zone. This study noted that this indicator should be omitted from the WQ protocol because animals raised in extensive tropical systems are pure or crossbred *B. indicus* genotypes that are characterized by having a more nervous temperament than *B. taurus* [21,32]. Furthermore, the grazing characteristic of this farming system facilitates little human/animal contact; therefore, the flight zone is not an indicator of animal welfare in this type of system. In intensive livestock farming, animals remain in the barn for most of their lives, but in extensive systems, this indicator, proposed by the WQ protocol, is not applicable because the animals are not kept confined in barns. In general, the WQ protocol assesses a total of 20 emotional states that, under extensive conditions, are very difficult to identify in animals. The recommendations shown in Table 1 were derived from the assessments conducted in the dry and rainy seasons.

## 5. Conclusions

Environmental variables provided better animal welfare conditions for cattle during the rainy season. The WQ protocol can be used as a basis for evaluating animal welfare, considering it important to make adjustments for its validation and application under semi-extensive conditions in the dry tropics. Some indicators are inapplicable to such local climate and rearing conditions; thus, they have been omitted, such as animal cleanliness, escape zone, access to grasslands (days per month, hours per day), and active, relaxed, nervous, aggressive, indifferent, happy, and frustrated temperaments. In contrast, some other indicators need to be added and should be further validated, such as the respiratory rate (gasping scale), shaded space per animal, presence of flies, branding, placement of earrings, weaning method, and clamping system, taking into account climatic variations between seasons to evaluate animal welfare more accurately.

## Figures and Tables

**Table 1:** Suggested modifications to the welfare quality protocol for its application in extensive production systems under tropical conditions.

Principle	Criteria	Indicators	Observations
Good feeding	1. Prolonged absence of hunger	1. Body condition	No modification
	2. Prolonged absence of thirst	2. Provision of sufficient water, clean drinking fountains, and clear water	Modification
Housing	3. Comfort at rest	3. Animal cleanliness	Omitted
	4. Thermal comfort.	4. Respiratory rate (gasping scale)	Added
	5. Ease of movement	5. Space per animal without shadow	No modification
		6. Shaded space per animal	Added
Health	6. Absence of injuries	7. Lameness	No modification
		8. Skin lesions	No modification
		9. Skin Inflammations.	No modification
	7. Absence of disease	10. Cough	No modification
		11. Nasal secretion	No modification
		12. Eye secretion	No modification
		13. Shortness of breath	No modification
		14. Diarrhea	No modification
		15. Tympany	No modification
	8. Absence of ectoparasites	16. Presence of flies	Added
	9. Absence of pain	17. Dehorning	No modification
		18. Branding	Added
		19. Weaning method	Added
		20. Placement of earrings	Added
		21. Clamping system	Added
		22. Butting	No modification
		23. Displacements	No modification
Behavior	10. Agonistic and social behavior	24. Fights	No modification
		25. Persecution	No modification
		26. Grooming	No modification
		27. Playing	No modification
	11. Human-animal relationship	28. Escape zone	Omitted
	12. Other behavior	29. Access to grasslands (days per month, hours per day.	Omitted
	13. Emotional state	30. Active, relax, nervous, aggressive, indifferent, happy, frustrated.	Omitted

**Table 2:** Average values of environmental variables recorded by season during the study year in the dry tropics, Guerrero state, Mexico.

Variables	Year Seasons				*p*-Value
	Dry Season		Rainy Season		
	Minimum	Maximum	Minimum	Maximum	
Temperature (°C)	32.9 ^a^	45.8 ^A^	23.4 ^b^	33.4 ^B^	<0.03
Relative humidity (%)	12.3 ^b^	25.3 ^B^	46.0 ^a^	90.2 ^A^	<0.001
* Rainfall (mm)	0.5 ^b^	8.5 ^B^	99.1 ^a^	561.2 ^A^	<0.0001
Temperature and humidity index (THI)	75.2–91.3		69.3–90.3		

* National Water Commission [16]. ^A,B^ and ^a,b^ letters’ different uppercase (maximum) and lowercase (minimum) in the same row indicate differences between seasons in the evaluation year (Mann–Whitney U test ≤ 0.05).

**Table 3:** Percentage grouping of bovine production units (n = 20) evaluated during the dry (DS) and rainy (RS) seasons according to animal welfare classification scales of the WQ protocol [7].

Scale (%) and Classification	Welfare Principles Scores							
	Good Feeding		Housing		Health		Behavior	
	DS	RS	DS	RS	DS	RS	DS	RS
≤20—Not Acceptable	100	55	-	50	20	-	100	25
>20–60—Acceptable	-	20	45	15	-	20	-	60
>60–80—Good	-	25	50	30	70	30	-	15
>80–100—Excellent	-	-	5	5	10	50	-	-

## Data Availability

Data are contained within this document. For any requests regarding data availability and clarifications, please contact the first author or the corresponding author.

## Abbreviations

The following abbreviations are used in this manuscript:

AW	Animal welfare
WQ	Welfare quality
PUs	Production units
DS	Dry season
RS	Rainy season
